# Controlled Release of H_2_S from Biomimetic Silk Fibroin–PLGA Multilayer Electrospun Scaffolds

**DOI:** 10.1021/acs.biomac.2c01383

**Published:** 2023-02-07

**Authors:** Anna Liguori, Elisabetta Petri, Chiara Gualandi, Luisa S. Dolci, Valentina Marassi, Mauro Petretta, Andrea Zattoni, Barbara Roda, Brunella Grigolo, Eleonora Olivotto, Francesco Grassi, Maria Letizia Focarete

**Affiliations:** † Department of Chemistry “Giacomo Ciamician” and INSTM UdR of Bologna, University of Bologna, Via Selmi, 2, 40126 Bologna, Italy; ‡ Interdepartmental Center for Industrial Research on Advanced Applications in Mechanical Engineering and Materials Technology, CIRI-MAM, University of Bologna, Viale Risorgimento, 2, 40136 Bologna, Italy; § RAMSES Laboratory, IRCCS Istituto Ortopedico Rizzoli, Via di Barbiano 1/10, 40136 Bologna, Italy; ∥ Health Sciences & Technologies (HST) CIRI, University of Bologna, Via Tolara di Sopra 41/E, 40064 Ozzano Emilia Bologna, Italy; ⊥ byFlow srl, Bologna 40129, Italy; # RegenHu Company, Z.I Du Vivier 22, CH-1690 Villaz-St-Pierre, Switzerland

## Abstract

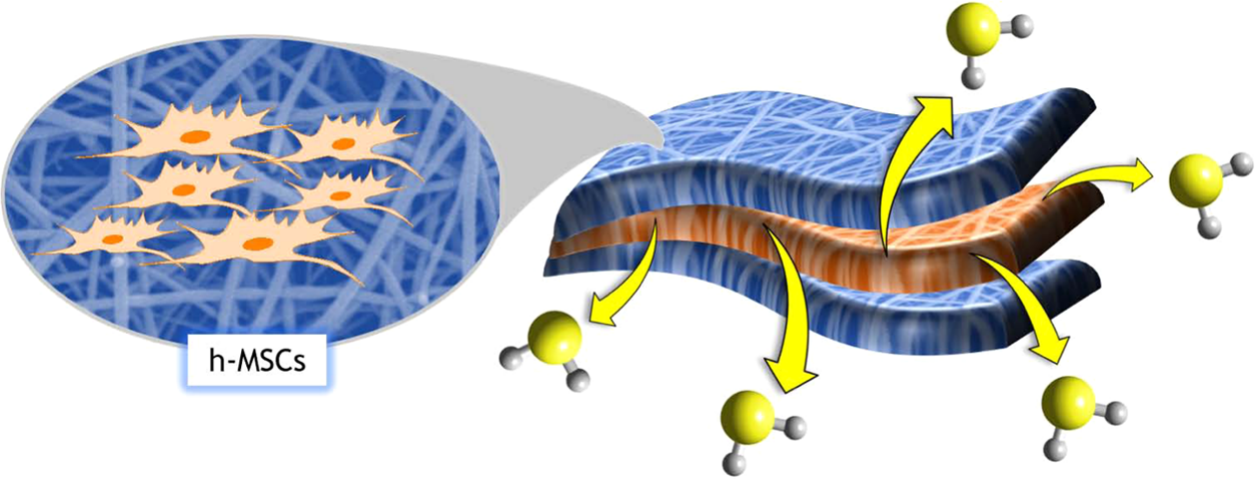

The possibility of incorporating H_2_S slow-release donors inside biomimetic scaffolds can pave the way to new approaches in the field of tissue regeneration and anti-inflammatory treatment. In the present work, GYY4137, an easy-to-handle commercially available Lawesson’s reagent derivative, has been successfully incorporated inside biomimetic silk fibroin-based electrospun scaffolds. Due to the instability of GYY4137 in the solvent needed to prepare silk fibroin solutions (formic acid), the electrospinning of the donor together with the silk fibroin turned out to be impossible. Therefore, a multilayer structure was realized, consisting of a PLGA mat containing GYY4137 sandwiched between two silk fibroin nanofibrous layers. Before their use in the multilayer scaffold, the silk fibroin mats were treated in ethanol to induce crystalline phase formation, which conferred water-resistance and biomimetic properties. The morphological, thermal, and chemical properties of the obtained scaffolds were thoroughly characterized by SEM, TGA, DSC, FTIR, and WAXD. Multilayer devices showing two different concentrations of the H_2_S donor, i.e., 2 and 5% w/w with respect to the weight of PLGA, were analyzed to study their H_2_S release and biological properties, and the results were compared with those of the sample not containing GYY4137. The H_2_S release analysis was carried out according to an “ad-hoc” designed procedure based on a validated high-performance liquid chromatography method. The proposed analytical approach demonstrated the slow-release kinetics of H_2_S from the multilayer scaffolds and its tunability by acting on the donor’s concentration inside the PLGA nanofibers. Finally, the devices were tested in biological assays using bone marrow-derived mesenchymal stromal cells showing the capacity to support cell spreading throughout the scaffold and prevent cytotoxicity effects in serum starvation conditions. The resulting devices can be exploited for applications in the tissue engineering field since they combine the advantages of controlled H_2_S release kinetics and the biomimetic properties of silk fibroin nanofibers.

## Introduction

Hydrogen sulfide (H_2_S) has been considered a toxic gas with a noxious odor for many years. Although the presence of H_2_S inside the mammalian tissues was already known, only in 1996 the endogenous production and signaling of this compound were elucidated, leading to its introduction into the family of gasotransmitters.^1^ H_2_S has been demonstrated to play a relevant role in regulating inflammatory processes, in the homeostasis of different tissues and organs,^2^ as a vasodilator substance,^3,4^ and in the regulation of angiogenesis and osteogenesis.^5,6^ In particular, H_2_S has been demonstrated to drive the capacity of self-renewal and multilineage differentiation into osteoblasts, chondrocytes, myocytes, and adipocytes of mesenchymal stromal cells (MSCs).^2^

Considering the therapeutic efficacy of H_2_S, several approaches have been investigated for its exogenous delivery, spanning from direct delivery methods (i.e., inhalation of gaseous H_2_S and introduction of sulfide salts, such as NaHS) to the use of H_2_S donors. Compared to the direct sources, the H_2_S donors enable a more controllable and prolonged release over time. Indeed, they need to be subjected to a chemical reaction under the action of a specific stimulus (i.e., water, light, pH, etc.) to release H_2_S. The control of the release kinetics using a reliable method is extremely important to modulate the stimulus correctly and avoid undesired toxic effects. Commonly employed methods to assess the release kinetics of H_2_S consist of (i) the use of selective electrochemical probes;^7,8^ (ii) the formation of methylene blue from the reaction of sulfide species in an acidic aqueous solution of *N*,*N*-dimethylphenylenediamine and iron (III) chloride (FeCl_3_);^9^ and (iii) the employment of selective fluorescent probes.^10,11^ In the past few years, there has been considerable interest in methods based on monobromobimane (MBB) for the derivatization and quantification of sulfide in solutions.^12−15^ However, complications related to the quantification of H_2_S can occur during laboratory procedures, mainly due to the quick oxidation of sulfide when exposed to air.^9^ Therefore, to avoid any undesired oxidation, strategies based on the use of chelating agents or the preparation of the solutions in the absence of oxygen are required.

Several synthetic H_2_S donors have been investigated over the years for therapeutic applications. Some of them have also been incorporated in hydrogels^16,17^ and polymeric scaffolds^18−22^ to achieve a long-lasting release of H_2_S, allowing local delivery at sites of tissue injury. Mauretti et al. proposed a poly(ethylene glycol)-fibrinogen hydrogel (PFHy) loaded with air or perfluorohexane-filled bovine serum albumin microbubbles coated with a TST enzyme able to catalyze H_2_S production.^16^ Wu et al. reported the development of a hyaluronic acid hydrogel doped with JK1, a hydrolysis-triggered pH-controllable donor, for dermal wound healing.^17^ This compound was also electrospun in a poly(caprolactone) (PCL) solution to obtain nanofibrous scaffolds showing a pH-dependent H_2_S releasing behavior for applications in wound dressing.^18^ Electrospun scaffolds of PCL and poly(l-lactic acid) for the release of *N*-(benzoylthio)benzamide derivatives and garlic-derived H_2_S donors, respectively, were also realized.^19^ In all of these studies, the methylene blue method was employed to determine the *in vitro* H_2_S release kinetics. Recently, Zhang et al. proposed the development of large porous microspheres (LPMs) containing a H_2_S-releasing aspirin derivative (ACS14), a novel synthetic H_2_S donor belonging to the family of dithiolthiones, for the treatment of pulmonary arterial hypertension. The H_2_S release kinetics was investigated *in vitro* by measuring the release of ACS14 and converting the moles of ACS14 into moles of H_2_S. The device was tested for the H_2_S release *in vivo* in rats’ lung tissue homogenates and plasma.^20^

Among H_2_S donors, the hydrolysis-triggered ones, i.e., Lawesson’s reagent derivatives and dithiolthiones, have been widely reported. GYY4137 (GYY) belongs to the class of Lawesson’s reagent derivatives, and it is the most commonly studied synthetic H_2_S donor, mainly due to its commercial availability and ease of handling. Furthermore, this compound is generally regarded as a slow-releasing H_2_S donor, showing release kinetics significantly lower than NaHS^23,24^ and a hydrolysis pathway occurring through a two-step process, as carefully described by Alexander et al.^25^ Focusing on the incorporation of GYY inside scaffolds, Patil et al. developed a nonaqueous *in situ* gelling sustained-release delivery system obtained by dissolving GYY in the poly(lactide-*co*-glycolide) (PLGA) solution prepared in a mixture of benzyl alcohol and benzyl benzoate.^21^ The use of a polymer soluble in nonaqueous solvents was necessary due to aqueous GYY instabilities to prevent compound’s hydrolysis. The resulting hydrogel, envisioned for the reduction of intraocular pressure in glaucoma pathogenesis, ensured a sustained release of H_2_S for 72 h, evaluated with the ethylene blue method, and did not show any significant toxicity. Raggio et al. developed silk fibroin sponges loaded with GYY by solvent casting and particulate leaching.^22^ GYY was incorporated using dimethyl sulfoxide as a vehicle. The H_2_S release kinetics was investigated employing an electrochemical method, using a sulfide gas amperometric microsensor. The results demonstrated that the scaffold did not induce cytotoxicity in any tested cells.

In the context of scaffolds for tissue engineering, the use of silk fibroin (SF) has been widely proposed for several applications. SF shows superior biocompatibility, controllable biodegradation, and attractive mechanical properties thanks to a balance of modulus, breaking strength, and elongation, which contributes to its toughness and ductility.^26^ Furthermore, SF scaffolds have been demonstrated to stimulate human osteoblast-like cell attachment, growth, and proliferation;^27^ to be suitable for tissue regeneration, including ligament, tendon, cartilage, bone, liver, skin, trachea, cornea, nerve, eardrum, and bladder;^28,29^ to be endowed with osteoinductive properties when loaded with recombinant human bone morphogenic protein-2 (rhBMP2).^30^ Among the native silk proteins, the silkworm silk, primarily that of the domesticated *Bombyx mori* (*B. mori*), has been recognized as a high-quality textile fiber and suture. The SF, obtained from *B. mori* silk fibers through specific extraction protocols,^31^ has also been widely employed to fabricate electrospun scaffolds for tissue engineering. Although some studies documented the possibility to electrospin SF from water solution,^4,32^ the majority of the works proposed the use of formic acid as a solvent^33−36^ since it enables the rapid solubilization of the SF obtained from the extraction process without inducing any degradation during the experimental period.^33^ In the context of bone tissue engineering, the use of electrospinning to produce scaffolds capable of stimulating osteogenesis finds a clinical application in guided bone regeneration (GBR), a procedure by which scaffolds are used at once to exclude non-osteogenic tissues from interfering with bone regeneration and to actively promote osteogenesis.^37^ Combining the osteogenic properties of H_2_S-releasing materials with biocompatible and clinically manageable scaffolds appears to be an attractive perspective to improve GBR-based bone regeneration.

Among the synthetic biodegradable and biocompatible polymers, PLGA is widely investigated and approved by the Food and Drug Administration (FDA) for therapeutic device development, spanning from sutures to tissue regeneration. PLGA is largely employed to obtain electrospun scaffolds for biomedical applications.^38^ The suitability of PLGA nanofibrous scaffolds as a drug delivery vehicle has also been documented, and it exploits the possibility of tailoring the nanofibers’ morphology by acting on process parameters to optimize the incorporation of drugs and their release from the nanofibers.^39,40^ The combination of PLGA and SF for obtaining electrospun scaffolds has allowed the development of invaluable materials for biomedical applications. In the context of wound treatment, the potentiality of PLGA/SF electrospun mats, produced with the technique of dual-source electrospinning, was investigated both *in vitro* and *in vivo*, confirming the most prominent wound healing effect of the bicomponent polymeric scaffolds compared to both the single components.^41^ The usefulness of this polymeric combination has also been investigated for bone tissue regeneration: PLGA/SF nanofiber scaffolds containing recombinant human bone morphogenetic protein-2 and dexamethasone were obtained via coaxial electrospinning. The devices were employed for in vitro bone formation with rat bone marrow mesenchymal stem cells and turned out to enable the sustained release of the two molecules, promoting cell adhesion and proliferation.^42^

In the present work, we have developed a biomimetic scaffold characterized by a nanofibrous and microporous morphology, able to release H_2_S in a controlled manner. The proposed scaffold has a multilayer architecture consisting of two external layers of electrospun silk to endow the scaffold with superior biomimetic properties and an internal layer of PLGA nanofibers incorporating GYY to achieve slow-release kinetics of H_2_S. The release of H_2_S was determined by applying an optimized analytical method based on the derivatization of MBB and high-performance liquid chromatography with fluorescence detection (HPLC-FLD). This method has shown high sensitivity and a limit of detection of 0.5 μM for the quantification of H_2_S species in serum samples,^43^ and it was successfully implemented for the kinetic study of H_2_S release.

## Materials and Methods

### Materials

Natural silk fibroin extracted from *Bombyx Mori* cocoon, purchased by Chul Thai Silk Co., Phetchabun, Thailand, was used. Poly(d,l lactide-*co*-glycolide) (PLGA), lactide/glycolide = 75:25 molar ratio (average molecular weight by GPC Mw = 50,900 g mol^–1^, polydispersity index, PDI = 1.55), was supplied by Evonik. H_2_S donor GYY (MW = 376.47 g mol^–1^), supplied as the morpholinium salt of (4-methoxyphenyl) morpholino-phosphinodithioic acid, was purchased from Cayman Chemical (Ann Arbor, MI). Formic acid (FA), dichloromethane (DCM), *N*,*N*-dimethylformamide (DMF), and ethanol (EtOH) were purchased from Sigma-Aldrich. Anhydrous sodium sulfide (Na_2_S, Sigma-Aldrich), monobromobimane (MBB, Sigma-Aldrich), tris(hydroxymethyl)aminomethane (Sigma-Aldrich), trifluoroacetic acid (TFA, Sigma-Aldrich), 5-sulfosalicylic acid dihydrate (SSA, Sigma-Aldrich), potassium dihydrogen phosphate (KH_2_PO_4_, Fluka Chemie GmbH), diethylenetriaminepentaacetic acid (DTPA, Sigma-Aldrich), and acetonitrile (Sigma-Aldrich) were used. Purified deionized water used throughout the study was obtained from a classic purification Millipore Milli-Q system (ELGA LC134, 0.2-micron filter, 18.5 mΩ cm^–1^, Milford, MA). Unless otherwise specified, all chemicals and solvents were used without further purifications.

### Preparation of Electrospun Solutions

#### Silk Fibroin Solution

Native *B. mori* cocoons were treated according to a previously reported protocol.^22^ Briefly, cocoons were degummed through two consecutive treatments in a 0.02 M Na_2_CO_3_ aqueous solution at 100 °C for 20 min each time. The obtained fibers were rinsed six times with warm ultrapure water and dried at 37 °C overnight. The degummed SF (Deg-SF) was dissolved at a concentration of 20% w/v in 9.3 M LiBr solution at 65 °C for 4 h. Afterward, the solution was dialyzed in a Slide-A-Lyzer Dialysis Cassette with 3500 MWCO (Thermo Fisher Scientific, Waltham, MA) against ultrapure water for two days, with regular water changes. The resulting silk fibroin solution was dried at 100 °C for 6 h, and a film was obtained. The electrospinning solution was finally obtained by dissolving the film in 98% v/v FA for 1 h at a concentration of 18% w/v.

#### PLGA/GYY solutions

PLGA/GYY solutions were prepared starting from a PLGA solution with a concentration of 33% w/v in DCM/DMF 25:75 v/v and GYY concentration of either 2% w/w or 5% w/w with respect to the weight of PLGA. The following procedure was employed: (i) GYY was dissolved in DMF, (ii) PLGA was slowly added to the solution, and (iii) after PLGA solubilization, DCM was added. The resulting solutions were kept for 20 min under stirring before electrospinning. PLGA solution, not containing GYY, was also prepared.

### Preparation of Electrospun Mats and Multilayer Scaffolds

Electrospun mats were prepared using an in-house electrospinning apparatus composed of a high-voltage power supply (Spellman, SL 50 P 10/CE/230), a syringe pump (KD Scientific 200 series, Massachusetts), a glass syringe, a stainless-steel blunt-ended needle connected with the power supply electrode, and a grounded steel plate collector (6.5 × 6.5 cm^2^). The entire system was located inside a glovebox (Iteco Eng., Ravenna, Italy, 100 × 75 × 100 cm^3^) equipped with a temperature and humidity control system. The polymer solution was dispensed through a Teflon tube to the needle orthogonally positioned with respect to the steel collector. The electrospinning of the silk fibroin solution was performed at a temperature and relative humidity of 25 °C and 30%, respectively, with a solution flow rate of 3 mL h^–1^, an applied voltage of 22 kV, and a gap of 20 cm between the needle outlet and the collector. Nanometric fibers with a random arrangement were collected, and the resulting silk fibroin mat was labeled SF-mat. The post-treatment of SF-mat was carried out by immersing it in absolute ethanol for 15, 30, and 60 min. Samples showing a thickness of around 135 μm and named SF-mat15, SF-mat30, and SF-mat60, respectively, were obtained.

Nonwoven PLGA/GYY mats were prepared from the two electrospinning solutions described above, containing different concentrations of GYY (2 and 5% w/w), and were labeled PLGA-2GYY and PLGA-5GYY, respectively. The electrospinning process was carried out at a temperature of 25 °C and a relative humidity of 40%, using an applied voltage of 20–22 kV, a solution flow rate of 6–8 mL h^–1^, and a gap between the needle and the collector of 20 cm. Electrospun scaffolds were kept under vacuum at room temperature (RT) for 30 min to remove residual solvents. Mats of plain PLGA, not containing GYY, were also produced as a reference and labeled PLGA. PLGA, PLGA-2GYY, and PLGA-5GYY showed a thickness of around 200, 190, and 120 μm, respectively.

Multilayer electrospun scaffolds were obtained by assembling a PLGA-based fibrous mat, sandwiched between two layers of SF-mat15, directly on a Scaffdex support (CellCrown24NX inserts). Multilayer samples containing GYY concentrations of 2 and 5% w/w were labeled ML2% and ML5%, respectively, whereas the reference multilayer sample without GYY is named ML0. Before biological experiments, the assembled scaffolds were sterilized using γ-rays (25 kGy).

### Chromatographic Method to Detect the Released H_2_S

The ad-hoc developed procedure for the H_2_S release study from the assembled scaffolds is schematically illustrated in Figure 4. The H_2_S-releasing tests were performed on multilayer scaffolds incubated in aqueous 0.1 M phosphate buffer (PB) (pH adjusted to 7.4 with 37% HCl at RT) in a thermostatic shaking bath at 37 °C. The H_2_S release was measured using the MBB method coupled with HPLC-FLD.^43^ The release of hydrogen sulfide in the incubation medium was monitored for 7 days at the following sampling time: 0, 2, 5, 19, 23, 28, 47, 72, 96, and 168 h. Three replicates for each sample were studied, and two aliquots for sampling time were examined to have statistical data. Quantitative determinations were carried out by peak area measurements at the emission wavelength of the SDB derivatization product after interpolation in a calibration curve prepared with Na_2_S standard solutions in PB (Supporting Information).

### Characterization methods

The morphological analysis of the electrospun mats was carried out using a Scanning Electron Microscope (SEM, Leica Cambridge Stereoscan 360) at an accelerating voltage of 20 kV. Prior to SEM analysis, the samples were sputter-coated with gold. The distribution of fiber diameters was determined through the measurement of about 300 fibers employing ImageJ software, and the results were given as the average diameter ± standard deviation. The Student’s unpaired *t*-test was used to test the statistical significance of the difference between the mean values (*p* < 0.05). Differential scanning calorimetry (DSC) measurements were carried out on SF-mat and SF-mat15 using Q2000 DSC (TA instruments, Delaware) in a N_2_ atmosphere from −90 to 250 °C, with a heating scan of 20 °C min^–1^. The glass transition temperature (Tg) was taken at half-height of the glass transition heat capacity step in the second heating scan performed after quenching. Thermogravimetric analysis (TGA) was conducted using a TA Instrument TGA Q500 analyzer in a N_2_ atmosphere by applying a temperature ramp of 10 °C min^–1^ from RT to 700 °C. Fourier transform infrared spectroscopy (ATR-FTIR) was carried out using a Spectrum Two (PerkinElmer) apparatus in attenuated total reflection mode. Each spectrum was collected in the wavenumber range 4000–400 cm^–1^, with a resolution of 4 cm^–1^ and 64 signal accumulations. Wide-angle X-ray diffraction (WAXD) analysis was performed directly on silk fibroin electrospun mats and SF-mat after the ethanol treatment deposited on quartz glass. The diffractogram patterns were recorded in the 5–60° 2θ range and a step rate of 0.03, with an X’Celerator detector at 40 and 40 kA, using a PANalytical X’Pert apparatus with a copper target and nickel filter.

### Biological Tests

#### Cells

Bone resident human MSCs (h-MSCs) were isolated, from the tibial plateau of 3 patients undergoing total knee replacement, after obtaining their informed consent, according to the procedure already established by the laboratory.^44^ Briefly, bone fragments were mechanically fragmented into small pieces to generate a cell suspension that was subjected to a Ficoll-density gradient isolation protocol, as previously reported.^44^ Cells were grown and expanded in α-MEM medium supplemented with 15% FBS and 1% penicillin/streptomycin until passage 2.

#### Live and Dead Staining

Cell viability of h-MSC seeded on the scaffolds was evaluated after 72 h in culture by the LIVE/DEAD^®^ Viability/Cytotoxicity Assay Kit (Thermo Fisher Scientific) based on the simultaneous determination of live (green) and dead (red) cells with two specific probes calcein-AM and ethidium homodimer (EthD-1), respectively. The scaffolds were washed with phosphate-buffered saline (PBS) and then incubated with ethidium homodimer 1 (4 μM) and calcein-AM (2 μM) for 30 min at 37 °C at 5% CO_2_. After two washing steps with PBS, the scaffolds were evaluated by an Eclipse 90i microscope equipped with Nikon Imaging Software elements (Nikon, Japan). For each sample, Z-stacking images at 10× magnification were captured. Z-stack images combine multiple images (28 consecutive layers) taken at different focal distances every 2.8 μm to provide a composite image for a total depth of 74.8 μm, as shown in Figure 7.

#### Cytotoxicity Assay

Quantification of cytotoxicity was performed using a colorimetric assay based on the measurement of lactate dehydrogenase (LDH) released in the supernatants by damaged cells, according to the manufacturer’s instructions (Cytotoxicity Detection Kit, Roche). h-MSCs were seeded onto the scaffolds at a concentration of 2 × 10^4^ cells/cm^2^ in α-MEM 15% FBS; after 24 h, the medium was replaced with α-MEM 15% FBS depleted of phenol-red for 72 h. At the end of incubation, 100 ml of supernatant was assayed for LDH release. Colorimetric detection of LDH was performed at 492–620 nm on a TECAN spectrophotometer, and cytotoxicity was calculated with reference to the control (unstimulated samples) and positive control (Triton X-100 treated samples) according to the formula
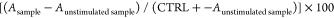
where *A* is the absorbance. Samples containing h-MSCs grown on plastic were used as a reference value. Each sample was assayed in triplicate.

#### Apoptosis (TUNEL Assay)

Quantification of cell death by apoptosis was performed by measuring the levels of cytoplasmic histone-associated DNA fragments (oligonucleosomes) through an ELISA assay, following the manufacturer’s instructions (Cell Death Detection Elisa Plus, Roche). Environmental stress was induced by culturing cells in the condition of serum starvation (5% FBS) for up to 72 h. h-MSCs were seeded onto the scaffolds, as stated above. After 24 h, the cells were starved by replacing the medium with α-MEM 5% FBS. A positive control (labeled as CTRL+ in Figure 7) is provided by the manufacturer, and it is constituted by a lyophilized DNA–histone complex; moreover, cells grown on the scaffold with α-MEM 15% FBS were used as a “negative” control for the occurrence of apoptosis. After 72 h in culture, cells were lysed, and apoptosis induced by reduced serum conditions was assessed in each sample by measuring the enrichment of nucleosomes in the cytoplasm. Colorimetric detection was finally performed at 405 nm on a TECAN spectrophotometer (Infinite M200). Each sample was assayed in triplicate.

## Results and Discussion

**Figure 1:**
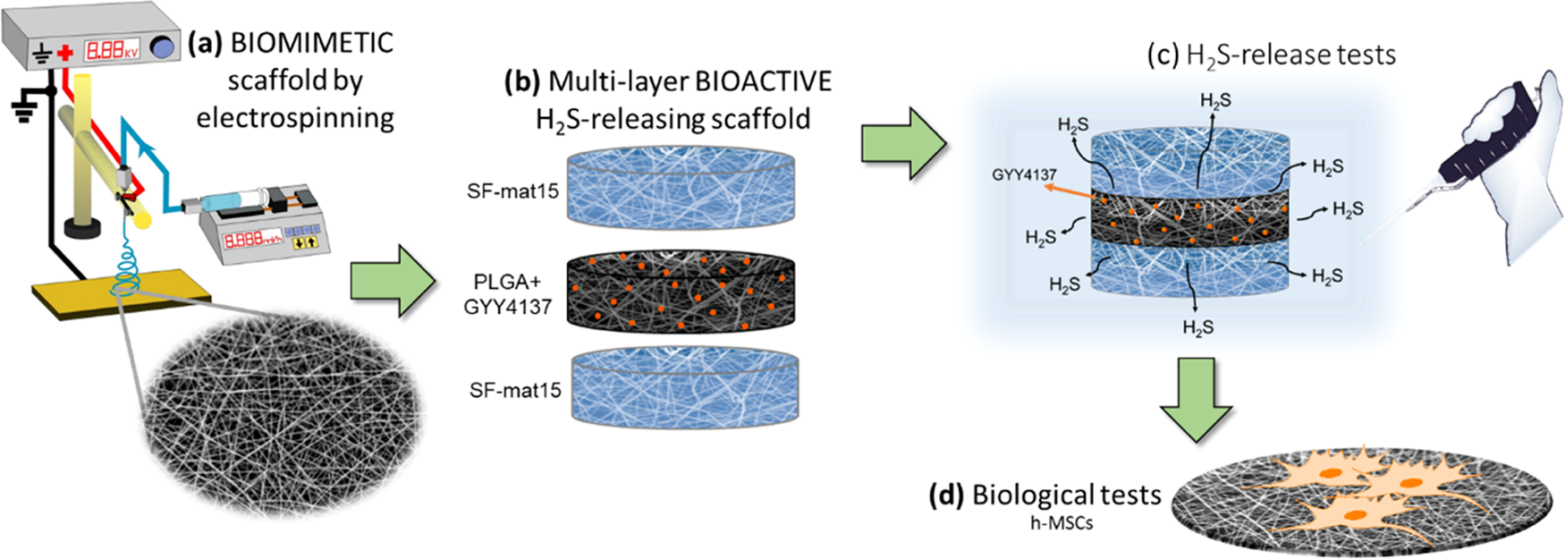
Schematic representation of the steps of the work: (a) production of fibrous biomimetic scaffolds of silk fibroin and PLGA by electrospinning, (b) preparation of multilayer bioactive scaffold releasing H_2_S, composed of an inner layer of PLGA loaded with GYY sandwiched between silk fibroin mats, (c) H_2_S release tests *in vitro*, and (d) h-MSC culture onto scaffolds.

This paper describes a new approach for developing a biomimetic SF electrospun scaffold loaded with the H_2_S-donor GYY molecule, intended to be used as a functional scaffold in tissue engineering applications (Figure 1). Among the native silk proteins, silkworm silk, mainly that of the domesticated *B. mori*, has been recognized as a high-quality textile fiber and suture. Furthermore, the immersion of the SF electrospun mat in ethanol or methanol solution has been widely demonstrated to induce a fast regeneration of the SF crystalline phase, which was lost during the extraction process.^33^

To overcome the instability of GYY in formic acid and in general in aqueous solutions,^24,25,45^ a multilayer electrospun scaffold composed of two external layers of electrospun SF and an inner layer of PLGA containing GYY was realized (Figure 1a,b). PLGA was selected in light of its biocompatibility and the possibility of being electrospun in organic solvents able to induce the solubilization of the H_2_S donor without causing its undesired hydrolysis. To investigate the effect of GYY concentration on the H_2_S release kinetics and on cells’ viability, two concentrations of the H_2_S release molecule (2% and 5% w/w with respect to the weight of PLGA) were introduced into the PLGA nanofibers.

**Figure 2:**
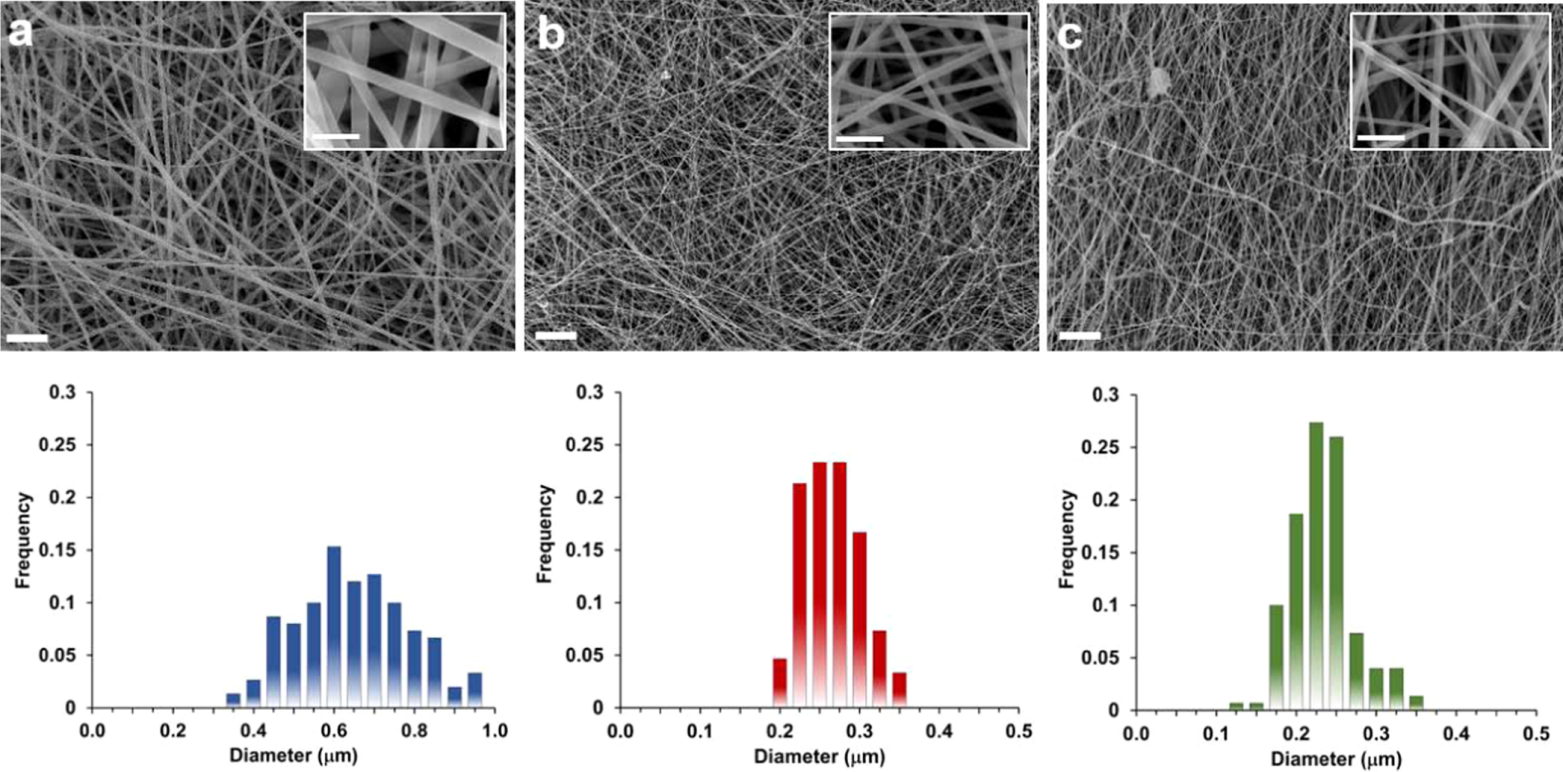
SEM images and corresponding fiber diameter distribution of PLGA and PLGA/GYY electrospun mats: (a) PLGA, (b) PLGA-2GYY, (c) PLGA-5GYY (scale bar = 10 μm). The insets show the SEM images at higher magnification (scale bar = 2 μm).

The morphological analysis of PLGA, PLGA-2GYY, and PLGA-5GYY (Figure 2) documented the possibility of electrospinning organic solutions of PLGA containing the H_2_S donor molecule. For both tested GYY concentrations, regular and bead-free nanofibers were obtained, whose diameter was influenced by the presence of GYY. Notably, the presence of the H_2_S releasing agent in the electrospinning solution enabled to obtain a reduction of the nanofibers’ mean diameter (277 ± 35 nm for PLGA-2GYY; 246 ± 40 nm for PLGA-5GYY) with respect to PLGA plain fibers (673 ± 140 nm), ascribable to an increase of the conductivity of the solution.

The influence of GYY on the thermal properties of the PLGA electrospun mats was investigated by DSC and TGA (Figure S1). As expected, the plain polymer is amorphous, with a glass transition around 50 °C and a marked enthalpic relaxation peak related to the polymer’s physical aging. The scaffolds show similar DSC curves, suggesting that the presence of GYY at the concentrations studied in this work does not affect PLGA thermal transitions. Similarly, TGA analysis shows that all PLGA-based samples degrade in a single step with a temperature of maximum weight loss rate (*T*_max_) of 300 °C (Figure S1), in line with literature findings.^46^ In contrast, GYY shows a more complex degradation behavior, with multiple steps and the main step at a *T*_max_ of 215 °C. GYY molecule does not affect the polymer degradation mechanism, but it seems to slightly increase PLGA stability: the polymer *T*_max_ goes from 300 °C for plain PLGA to 312 °C for PLGA-2GYY and to 326 °C for PLGA-5GYY. From the analysis of the vibrational absorbance peaks in the FTIR/ATR spectra, no signals due to the GYY molecule were observed, in addition to the expected signals of PLGA, due to the low amount of this component inside the PLGA fibers (Figure S1).

**Figure 3:**
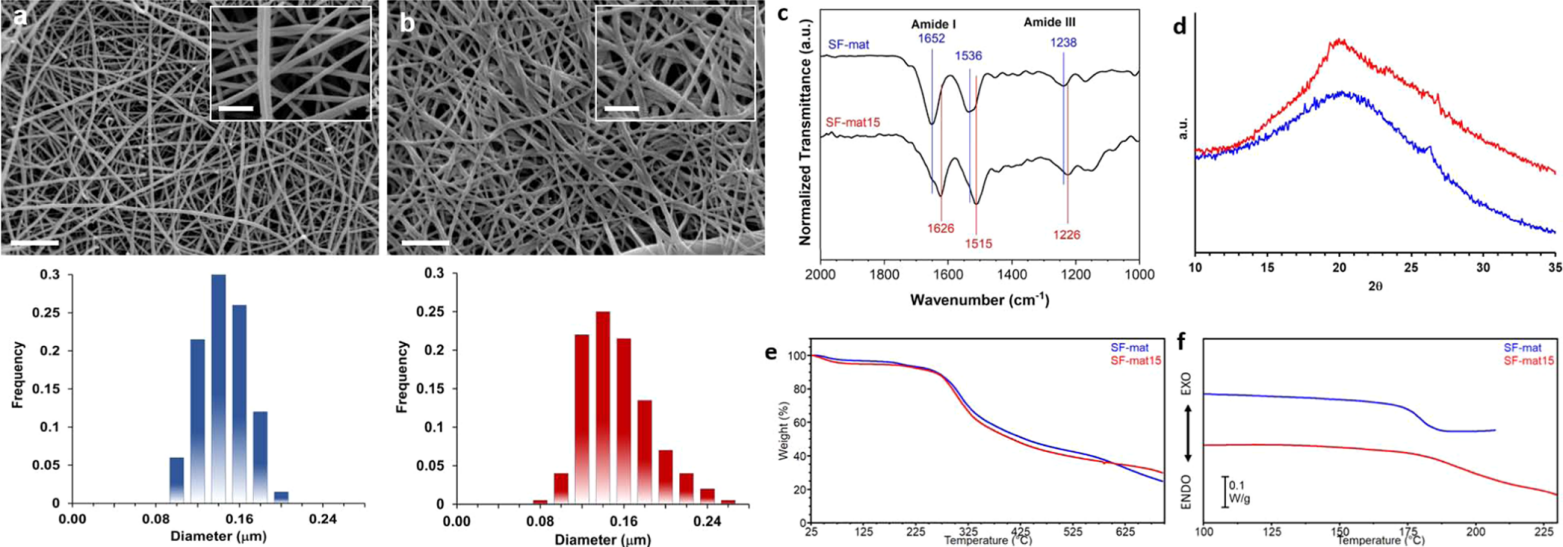
SEM images and corresponding fiber diameter distribution of (a) the SF electrospun mat and (b) SF-mat15 electrospun mat. Scale bar = 2 μm. Insets: scale bar = 1 μm. (c) ATR-IR spectra, (d) WAXD, (e) DSC curves (heating scan after quenching), and (f) TGA curves of SF and SF-mat15 mats.

In line with previously reported studies,^33,34,47^ the electrospinning of SF in formic acid enabled to obtain regular nanofibers, as evident in Figure 3a, with a mean diameter of around 145 ± 23 nm. To stabilize the structure of SF by inducing the transition of conformation from random coil to crystallizable β-sheet, the SF-mats were subjected to ethanol treatment. In fact, the amorphous structure of SF-mats causes the material to be water soluble and can limit its applications in aqueous biological environments. The immersion of the SF-mat in ethanol solutions has been demonstrated to induce a fast regeneration of the SF crystalline phase, which was lost during the extraction process.^48^ As shown in Figure 3b, the treatment performed by immersing the samples for 15 min in absolute ethanol turned out not to significantly affect the fibers’ morphology and mean diameter (163 ± 32 nm), differently from longer immersion times (i.e., 30 and 60 min) for which a partial swelling of the fibers and partial loss of mats’ porosity were registered (Figure S2). ATR-IR spectra were collected to investigate the vibrational mode of SF-mat and SF-mat15 amides, which are correlated to the organization of the secondary structure of the protein. Figure 3c documents a significant shift of the SF’s amide I and amide III absorption peaks to lower wavenumbers after the treatment in ethanol, clearly highlighting the occurrence of the transition from α-helix/random coil to β-sheet conformation.^48−50^ WAXD analysis (Figure 3d) was in line with previously reported studies^33,49^ and highlighted the presence in the SF-mat of silk I, which accounts for the amorphous structure of the fibroin, as documented by the broad peak centered at 2θ = 22°. After the ethanol treatment, a sharper peak at 19°, followed by a slightly detectable peak at 24°, documented the induction of the β-sheet crystalline phase,^49,51,52^ although the amorphous phase still resulted being the most prevalent one. DSC analysis was also performed to investigate further the ethanol treatment’s effect on the SF macromolecule conformation (Figure 3e). Due to the limited thermal stability of the SF-mats, demonstrated by the TGA analysis carried out on SF-mat and SF-mat15 (Figure 3f), the DSC heating scans were carried out only up to around 220–250 °C to highlight the endothermic step associated with the glass transition. The calorimetric curves, reported in Figure 3e, document the shift of the SF *T*_g_ toward higher temperature after the ethanol treatment, accounting for an increase in the mat’s rigidity and confirming an increase of the more stable β-sheet conformation in SF-mat15. Taken altogether, the results of the solid-state characterization of SF electrospun mats confirmed the hypothesis that the ethanol post-treatment induced the formation of the β-sheet conformation in the SF secondary structure, in addition to the α-helix/random coil phase that continued to be the most prevalent phase.

**Figure 4:**
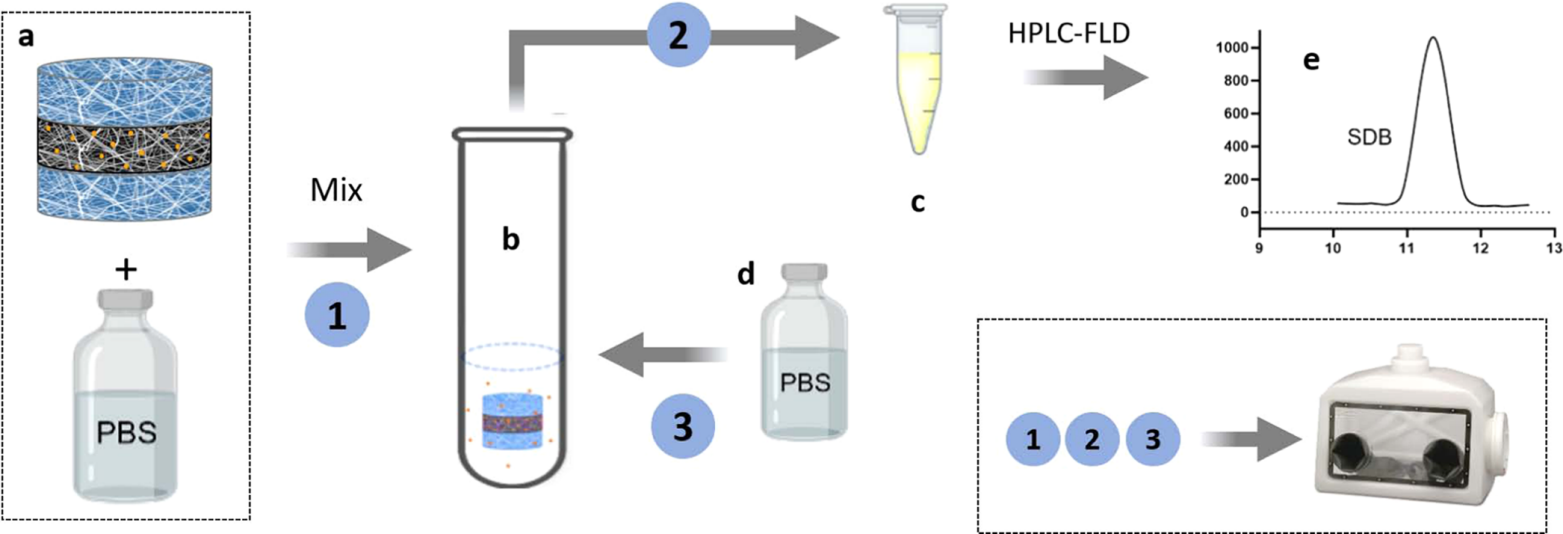
Schematic representation of the H_2_S quantification method: (a) 4 mL of PB, sonicated for 30 min before use to remove dissolved gasses and/or entrained gas bubbles, and the multilayer sample was placed into a 20 mL plastic tube first; (b) afterward, the tube was wrapped with an aluminum film and incubated in the thermostatic shaking bath at 37 °C; (c) derivatization reaction of releasing H_2_S with the fluorescent alkylating agent MBB; (d) after each withdrawal, the PB solution was refilled with the same amount of PB (30 μL); (e) quantification of hydrogen sulfide: derivatization of H_2_S with MBB, forming a sulfide-dibimane (SDB) product via S-alkylation; the resultant fluorescent SDB is analyzed by HPLC-FLD; the H_2_S concentration was then obtained after SDB area interpolation in a calibration curve (Supporting information). Steps 1, 2, and 3 were carried out in a hypoxic chamber (1% O_2_) at RT.

The electrospun SF and PLGA/GYY mats were assembled into a multilayer architecture (Figure 1) and sterilized before being subjected to H_2_S release studies. As is well known, the unstable nature of free hydrogen sulfide in solution makes measurement and analysis usually difficult. In this work, the MBB method with HPLC-FLD, suitable for sensitive, quantitative measurement of hydrogen sulfide, was employed according to the ad-hoc developed procedure schematically illustrated in Figure 4.

**Figure 5:**
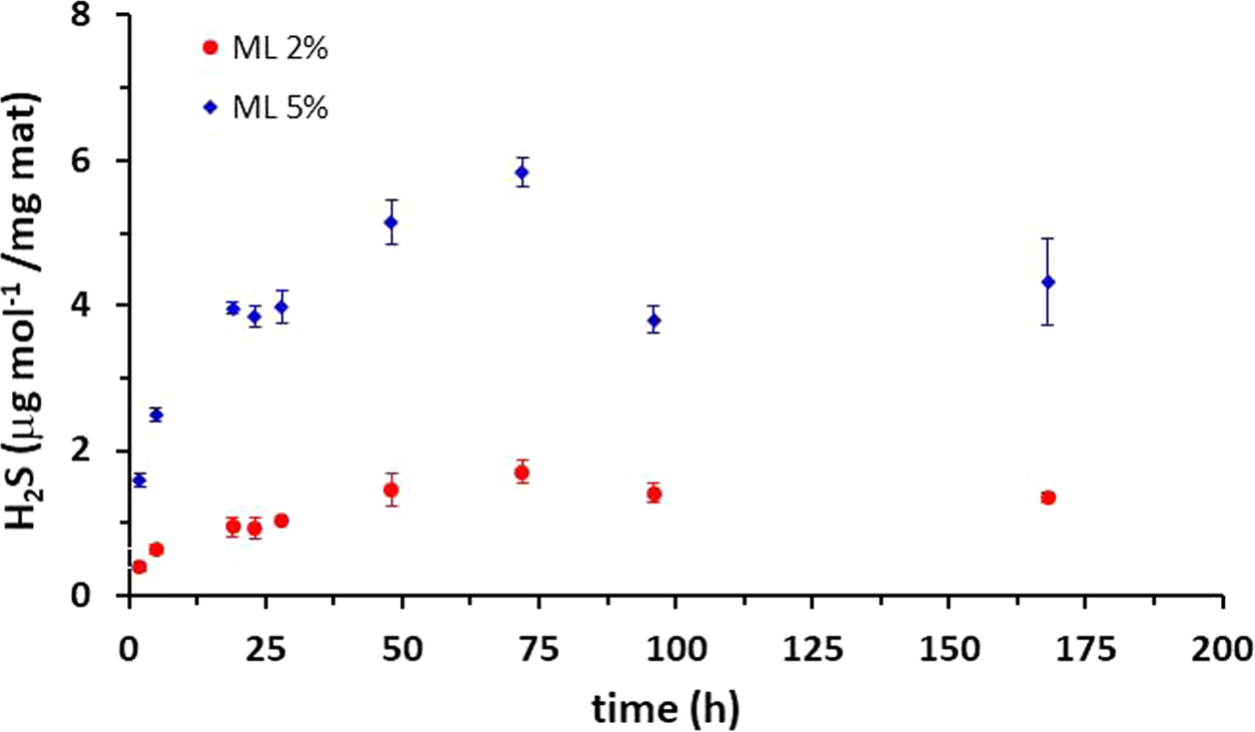
H_2_S-release from sterilized multilayer samples from 0 to 168 h. Three replicates for each kind of sample were assembled, and two derivatizations for each assembled sample were tested and analyzed by HPLC-FLD. Each point is the result of the measurement of six different concentrations. The values plotted represent mean ± SD.

The HPLC-FLD procedure (see Material and Methods) was first applied to GYY in PB solutions (see the Supporting Information) to check the instrumental setup and verify the efficacy of GYY molecule as a H_2_S donor in the solution (Figure S4a,b). Results show an increase of H_2_S release in 5h, and then values remain almost stable. A slight decrease, probably ascribable to the degradation of H_2_S in the medium, can be observed at time points higher than 20 h. Subsequently, we tested three replicates of multilayer scaffolds without GYY (ML0). No signal was obtained after the derivatization step and HPLC-FLD analysis for all sampling time points (data not shown). Conversely, signals were observed for multilayer samples ML2% and ML5%, assembled with different amounts of GYY in the PLGA layer (0.08 mg for ML2% and 0.25 mg for ML5%), highlighting controlled kinetics for H_2_S release (Figures 5 and S5). Results show that the sulfide concentration presents an increasing trend and reaches the highest value in 72 h for both ML2% and ML5%. On the other hand, a slow decrease in H_2_S concentration was detected after 72 h of immersion, as already shown in previous studies investigating the H_2_S release from donors embedded in the polymeric matrix.^18,44^ Furthermore, in tune with previous work,^44^ both the increase and decrease of the sulfide concentration at shorter and longer releasing times, respectively, turned out to strongly depend on the concentration of GYY loaded in the scaffold.

The obtained results can be compared with those reported by Patil et al.^21^ in their work dealing with the H_2_S release from a PLGA/GYY gelling delivery system thought for the treatment of glaucoma pathogenesis. The device was characterized by a GYY/PLGA weight ratio of 2% w/w, and it turned out to enable a sustained H_2_S release in simulated tear fluid. Indeed, after 24 h, the cumulative release of H_2_S from the formulation was around 5 μg mL^–1^. Regarding our scaffolds, the release of H_2_S from ML2% and ML5% after 20 h of immersion in PB were 0.19 and 1.02 μg mL^–1^, respectively. The differences in the released H_2_S concentration might be explained by considering the greater propensity of gel formulations to swell and release molecules in a liquid environment in comparison to the solid nanofibrous scaffold reported in our work. It is worth noting that with respect to the solid porous SF devices containing GYY proposed by Raggio et al.,^22^ both the ML scaffolds (2 and 5%) demonstrated comparable GYY encapsulation efficiency and amount of released H_2_S after around 2 h of immersion in an aqueous solution. Moreover, while in Raggio et al. work,^22^ the H_2_S release reached the plateau after 90 min of immersion for all of the tested GYY concentrations, the ML scaffolds described in the present work are characterized by a more controllable and sustained release over time, thus representing a valuable platform to modulate the H_2_S release kinetics. When compared to similar scaffolds based on electrospun fibers, our system shows a comparable or more sustained H_2_S release: Cacciotti et al.^56^ described a fibrous mat based on electrospun PLA with H_2_S releasing capacity, but in this system, the H_2_S release peaked after 2 h of incubation in an aqueous medium. Feng et al.^19^ described a construct based on electrospun PCL fibers and showed that the diameter of the fibers significantly affects H_2_S release; even in this system, the release of H_2_S reached a plateau after nearly 24 h, and, in the following 70 h, it slowly declined. Importantly, consistent with our findings, in each of these works, a scaffold obtained with micro or nanostructured electrospun fibers appears to establish a pro-regenerative microenvironment by counteracting oxidative cell damage, favoring cell colonization and viability and supporting the neosynthesis of extracellular matrix components.

**Figure 6:**
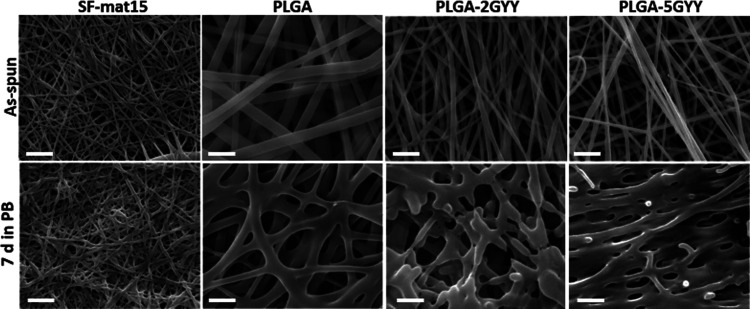
SEM images of SF-mat15, PLGA, PLGA-2GYY, and PLGA-5GYY as produced (upper row) and after 7 days of H_2_S-releasing tests (lower row). Scale bar = 5 μm.

To better understand the results obtained from the H_2_S release tests, SEM analyses were carried out on each layer of the three multilayer scaffolds after 7 days of immersion in PB, and the results were compared with those obtained on the layers not subjected to the release tests. Figure 6 reports the good preservation of SF-mat15 after the release test, documenting that the morphology of the external layers is not compromised by the H_2_S release from the inner layer. Conversely, PLGA fibers significantly change their morphology after PB immersion, showing increased fiber diameter and “fusion” at contact points. This swelling effect is even more evident with the increase of GYY concentration, highlighting the increased capability of PLGA nanofibers to be penetrated by PB in the presence of the H_2_S donor inside the fibers. The absorption of the aqueous solution inside the fibers might drive the release of H_2_S from the multilayer scaffolds.

**Figure 7:**
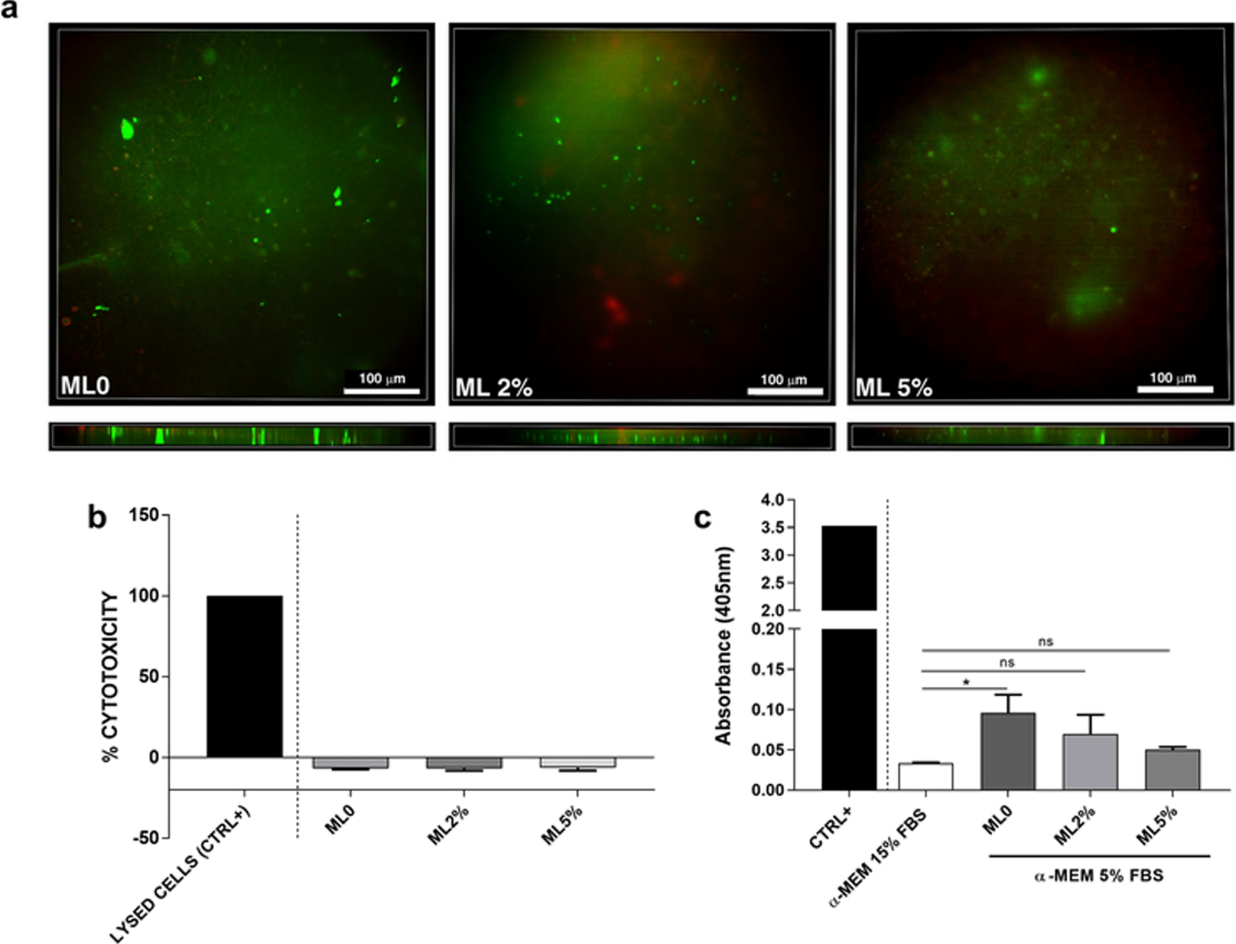
(a) Cell viability evaluated by Calcein-AM/Ethidium homodimer staining. Human MSCs were seeded on the scaffolds and stained after 72 h in culture. Panels show a top-view, single-layer picture, and a picture representing the transversal section of each scaffold for a total depth of 78.4 μm across each scaffold. (b) Cytotoxicity assay: LDH released in the supernatants by h-MSC was measured after 72 h in culture. Data represent the % cytotoxicity using cells grown on plastic as a control (negative) sample and are expressed as mean ± SD of *N* = 3 independent experiments. (c) Histograms representing the enrichment of cytoplasmic oligonucleosomes in h-MSC after 72 h of starvation. Data are expressed as mean ± SD of *N* = 3 independent experiments. *p < 0.05 compared to control cells grown on α-MEM 15% FBS; CTRL+ represents a positive control provided by the manufacturer, constituted by lyophilized DNA–histone complex. ns = not significant.

To evaluate the effect of the electrospun mats with and without GYY on a relevant biological system, h-MSCs were cultured onto the scaffolds for up to 72 h, and cytotoxicity and cell viability were assayed both in standard conditions and in starvation, maintaining MSCs in medium containing 2% FBS. Cellular viability after 72 h in culture was first assessed through the vital dyes calcein and propidium iodide; Figure 7a shows representative pictures of h-MSCs at the end of culture in each of the tested multilayer scaffolds, namely, ML0, ML2%, and ML5%. The 3D-stack images (Figure S6) of the scaffolds show that cells are similarly distributed across the three dimensions and are mostly alive in each sample. Furthermore, quantification of the LDH assay performed after 72 h confirmed that the mats are completely devoid of cytotoxicity even in the presence of GYY at 2 or 5%. In Figure 7b, the histogram reports the values of LDH release referred to cells grown on plastic as a control and shows that cytotoxicity remained below this reference value in each mat. To evaluate the capacity of H_2_S-releasing devices to protect cells against deadly stress, we performed a set of experiments by culturing h-MSCs in conditions of reduced serum (5%) for up to 72 h. Serum starvation is one way to establish environmental stress, which may lead to cell death by activating various pathways.^53,54^ When serum starvation was maintained for 72 h, it resulted in a significant increase of apoptosis in MSC grown on ML0 mats, where no H_2_S is released, compared to the control sample represented by MSC grown on α-MEM 15% FBS (Figure 7c); increased apoptosis of MSC in the condition of serum starvation is in agreement with previous reports.^55^ Conversely, the increased level of apoptosis was prevented in mats containing 2 and 5% GYY, suggesting an active role of H_2_S in preventing cell death. This result can be ascribed to the widely documented potential of H_2_S-releasing biomaterials to provide a healing environment at sites of tissue damage.^19,56,57^

## Conclusions

Silk fibroin-based electrospun scaffolds for the controlled release of H_2_S and suitable for tissue engineering applications were successfully realized. The scaffolds were characterized by a multilayer architecture, in which a PLGA nanofibrous layer containing GYY was sandwiched between two silk fibroin nanofibrous mats, previously treated in EtOH to favor the formation of the crystalline phase. Morphological analysis carried out on the PLGA layer documented a decrease in the fibers’ mean diameter, with the increase of GYY concentration attributed to an increase in the polymeric solution’s conductivity in the presence of this molecule. From thermal analysis, GYY contributed to a slight increase in the PLGA thermal stability without affecting its glass transition temperature. No relevant modifications of the ATR-FTIR spectrum of PLGA were observed in the presence of the H_2_S donor due to its low concentration inside the electrospun nanofibers. SEM analysis of the silk fibroin electrospun mats confirmed the formation of regular and homogeneous nanofibers, whose morphology was not relevantly affected by the 15 min post-treatment in EtOH. The solid-state characterization of the silk fibroin electrospun mats performed through ATR-FTIR, WAXD, and DSC confirmed the formation of the β-sheet conformation in the SF secondary structure after the 15 min EtOH post-treatment, in addition to the α-helix/random coil. The investigation of the H_2_S release kinetics from the multilayer scaffolds, performed in PB according to a properly designed procedure, documented a controlled delivery over 168 h and the possibility of modulating it by acting on GYY concentration inside the PLGA layer. The highest release registered for ML5% with respect to ML2% lies in the more evident “swelling” effect observed for the scaffolds with the increase of GYY concentration. In this context, future studies will be devoted to investigating possible strategies to prevent this partial loss of SF-mats fibrous morphology for long-time immersion in an aqueous solution, also with the aim to further optimize the kinetics of H_2_S release.

When cells were added to the device, it became apparent that this multilayered scaffold supports cell colonization and spreading with no evident sign of toxicity linked to H_2_S release. Moreover, in keeping with the cytoprotective role of H_2_S, we found that H_2_S-releasing devices were able to mitigate the cytotoxic effect induced by prolonged serum starvation as compared to control mats. The obtained *in vitro* results support the potential role of this scaffold in maintaining cell integrity and promoting tissue regeneration and pave the way for the use of H_2_S-releasing mats in procedures of GBR-based bone tissue regeneration and for future *in vivo* studies of tissue damage, also in different biological tissues.

## Supporting Information Available

Characterization of electrospun PLGA/GYY4137 scaffolds; optimization of the ethanol treatment on SF-mats and characterization; preparation of the calibration curve for sulfide-dibimane (SDB) quantification; degradation of GYY and H_2_S release; and cell viability evaluation (PDF)
