# Supplementary material for: Controlled Release of H2S from Biomimetic Silk Fibroin–PLGA Multilayer Electrospun Scaffolds
Source: Biomacromolecules. 2023 Feb 7;24(3):1366–76. doi: 10.1021/acs.biomac.2c01383 (PMC10015463; doi:10.1021/acs.biomac.2c01383)
Supplement: Supplementary file 1 — bm2c01383_si_001.pdf [file bm2c01383_si_001.pdf]

Supporting Information

Controlled release of H_2S from biomimetic Silk Fibroin-PLGA multi-layer electrospun scaffold

Anna Liguori[a‡], *Elisabetta Petri*[a‡], *Chiara Gualandi*[a,b,d], *Luisa S. Dolci*[a], *Valentina Marassi*[a,e], *Mauro Petretta* [c,f], *Andrea Zattoni*[a,e], *Barbara Roda*[a,e], *Brunella Grigolo*[c], *Eleonora Olivotto*[c], *Francesco Grassi*[c], *Maria Letizia Focarete*[a,d*]

[a] Department of Chemistry "Giacomo Ciamician" and INSTM UdR of Bologna, University of Bologna, Via Selmi, 2, 40126 Bologna, Italy
[b] Interdepartmental Center for Industrial Research on Advanced Applications in Mechanical Engineering and Materials Technology, CIRI-MAM, University of Bologna, Viale Risorgimento, 2, 40136 Bologna, Italy
[c] RAMSES Laboratory, IRCCS Istituto Ortopedico Rizzoli, Via di Barbiano 1/10, 40136 Bologna, Italy.
[d] Health Sciences & Technologies (HST) CIRI, University of Bologna, Via Tolara di Sopra 41/E, 40064 Ozzano Emilia Bologna, Italy
[e] byFlow srl, Bologna, Italy
[f] RegenHu Company, Z.I Du Vivier 22, CH-1690 Villaz-St-Pierre, Switzerland

[±]equally contributed
*marialetizia.focarete@unibo.it; Department of Chemistry "Giacomo Ciamician" and INSTM UdR of Bologna, University of Bologna, Via Selmi, 2, 40126 Bologna, Italy

Contents

1. Characterization of electrospun PLGA/GYY scaffolds

2. Optimization of the Ethanol treatment on SF-mats and characterization

3. Preparation of calibration curve for sulfide-dibimane (SDB) quantification

4. Degradation of GYY4137 and H_2S release

5. Cell viability evaluation

1. Characterization of electrospun PLGA/GYY scaffolds

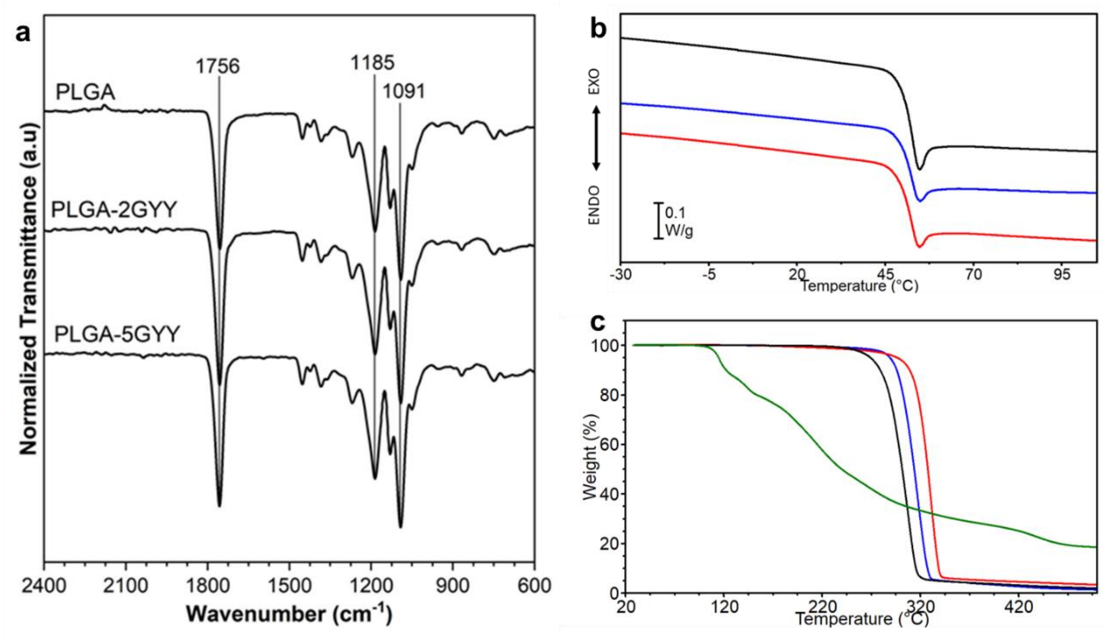

Figure S1. (a) ATR-FTIR spectra of PLGA electrospun mats: PLGA; PLGA-2GYY; PLGA-5GYY (absorption bands characteristic of PLGA are indicated). (b) DSC curves (heating scan after quench) and (c) TGA curves of: PLGA (black), PLGA-2GYY (blue), PLGA-5GYY (red), and GYY powder (green).

2. Optimization of the Ethanol treatment on SF-mats and characterization

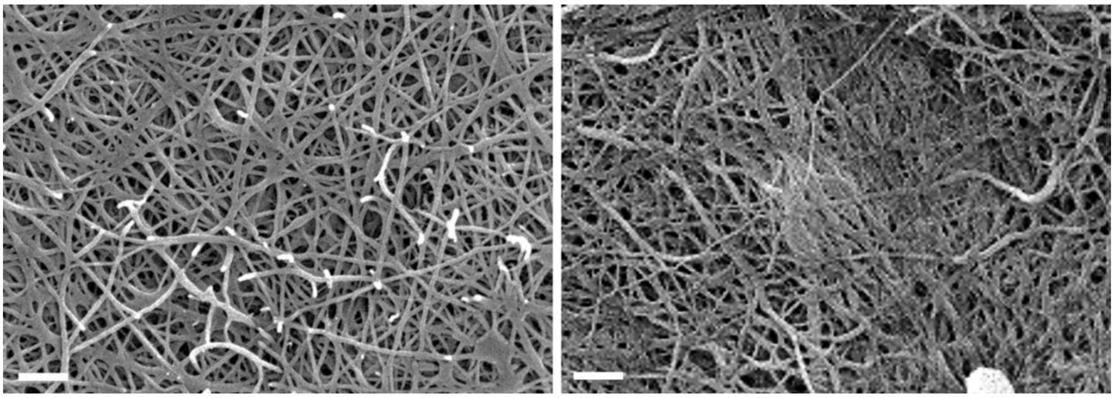

Figure S2. SEM images of silk fibroin electrospun mats after 30 minutes (a) and 60 minutes (b) of EtOH treatment. Scale bar = 2 μm. Insets: scale bar = 1 μm.

3. Preparation of calibration curve for hydrogen sulfide quantification

Solutions:

PB: Phosphate buffer (0.1 M, pH adjusted with HCl)

Tris-HCl buffer (pH 9.5, 0.1 mM DTPA)

Monobromobimane (MBB) solution: 1.5 mM in Acetonitrile, the solution should be kept in an amber container and protected from light to avoid photolysis.

Sulfosalicylic acid dihydrate (SSA) solution: 200 mM in water

MBB-HPLC-FLD quantification of H_2S

The MBB method mainly consisted of (a) derivatization, (b) HPLC injection and (c) FLD detection. The first step consisted of: (i) withdrawal of 30 µL of the solution in which the samples were incubated; (ii) addition of 70 µL of Tris-HCl buffer (100 mM, pH=9.5, 0.1 mM DTPA); (iii) addition of 50 µL of 1.5 mM MBB solution (5 mL Acetonitrile + 2.0 mg MBB); (iv) reaction incubation for 30 minutes at 50 °C; (v) reaction quenching with 50 µL of 200 mM 5-sulfosalicylic acid (SSA). PB additions and all withdrawals were performed in a hypoxic chamber (1% O_2). The entire derivatization procedure was carried out under dim light.

Sodium sulfide (Na_2S) was used as a source of hydrogen sulfide for standard solutions. A 12.8 mM stock solution in PB was freshly prepared and stored in a 10 mL opaque centrifuge tube at RT. Six final concentrations of calibration standards (1, 5, 10, 15, 25, and 40 µM H_2S) were then prepared by diluting the stock solution with PB. All working standard solutions were freshly prepared for derivatization every day.

Then, 30 µL of the obtained derivatized sulfide dibimane (SDB) samples were injected into HPLC using a flow rate elution of 0.6 mL min^{-1}, and a binary mixture comprising a mobile phase A (water) and a mobile phase B (acetonitrile), adjusted with 0.1% (v/v) trifluoroacetic acid (TFA) employing the HPLC-FLD method already reported [*B. Roda, N. Zhang, L. Gambari, B. Grigolo, C. Eller-Vainicher, L. Gennari, A. Zappi, S. Giordani, V. Marassi, A. Zattoni, P. Reschiglian, F. Grassi, Optimization of a Monobromobimane*

(MBB) Derivatization and RP-HPLC-FLD Detection Method for Sulfur Species Measurement in Human Serum after Sulfur Inhalation Treatment, Antioxidants,11(5) (2022) 939.]. Excitation and emission wavelengths of the fluorescence detector were set at 390 and 475 nm, respectively.

The calibration curve was obtained through a polynomial regression analysis by plotting the FLD peak area of SDB against the H_2S standard concentration values (Figure S3). Equation:

$y = 0.013x^2 + 10.87x - 5.24$, $R^2 = 0.999$.

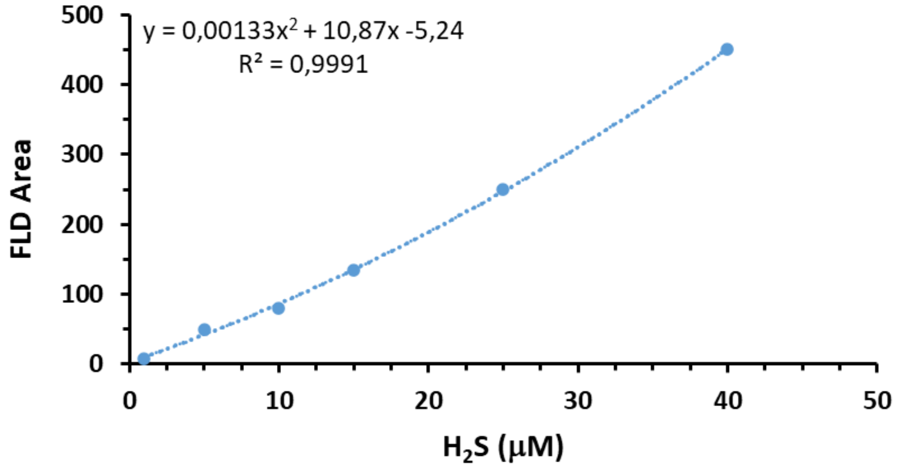

Figure S3. Calibration curve for hydrogen sulfide quantitation.

4. Degradation of GYY and H_2S release

The GYY (0.38 mg) was placed in a 2 mL Eppendorf PCR tube with a tight cap, then 1 mL of PB (0.1 M, pH= 7.4) was added. The tube was incubated in the tilting bath at 37°C for 168 h.

An aliquot of 1 mL was used as blank sample. Sampling was carried out in a hypoxic chamber on GYY and blank; after each withdrawal, the PB solution was refilled with the same amount of PB (30 µL). SDB levels were quantified after 30 min derivatization in hypoxic chamber with 1.5 mM MBB in the dark at pH = 9.5 in 100 mM Tris-HCL buffer at 50 °C. The resultant fluorescent SDB was analysed by HPLC-FLD. The normalized levels of sulfide (µg mol^{-1}/mg GYY) were calculated through the

interpolation of SDB Area in the calibration equation.

(a)

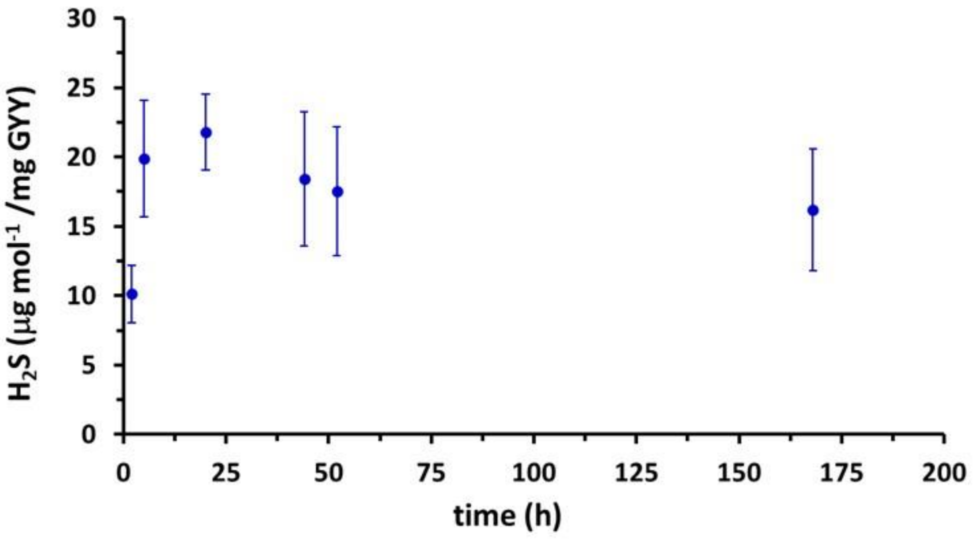

(b)

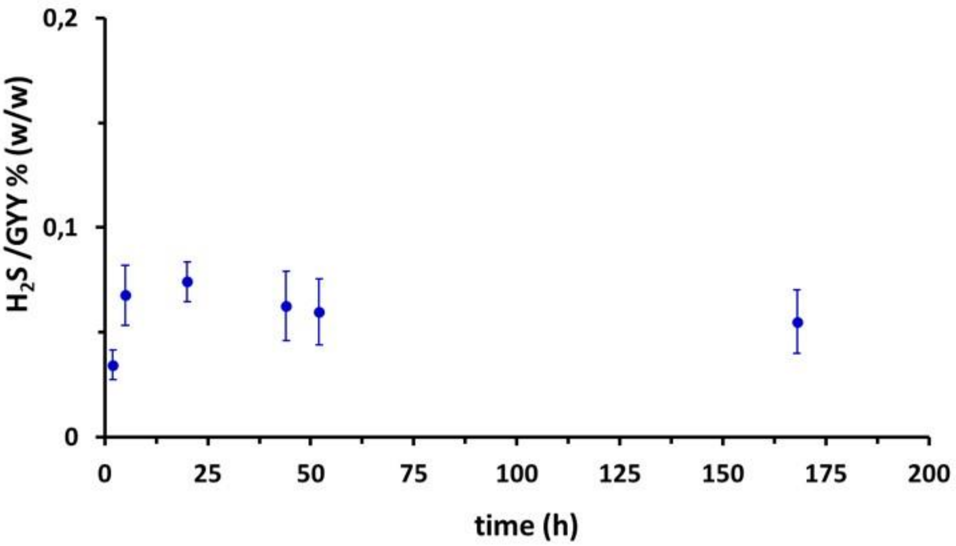

Figure S4. H_2S-releasing of GYY from 0 h to 168 h (timepoints: 0, 2, 5, 20, 44, 52, and 168 h) with respect to the amount of GYY: a) (μg $mol^{-1}$of H_2S) / (mg of GYY); b) (μg of H_2S/μg of GYY)%. Data points represent the average values of n = 4 measurements with error bars showing standard deviation.

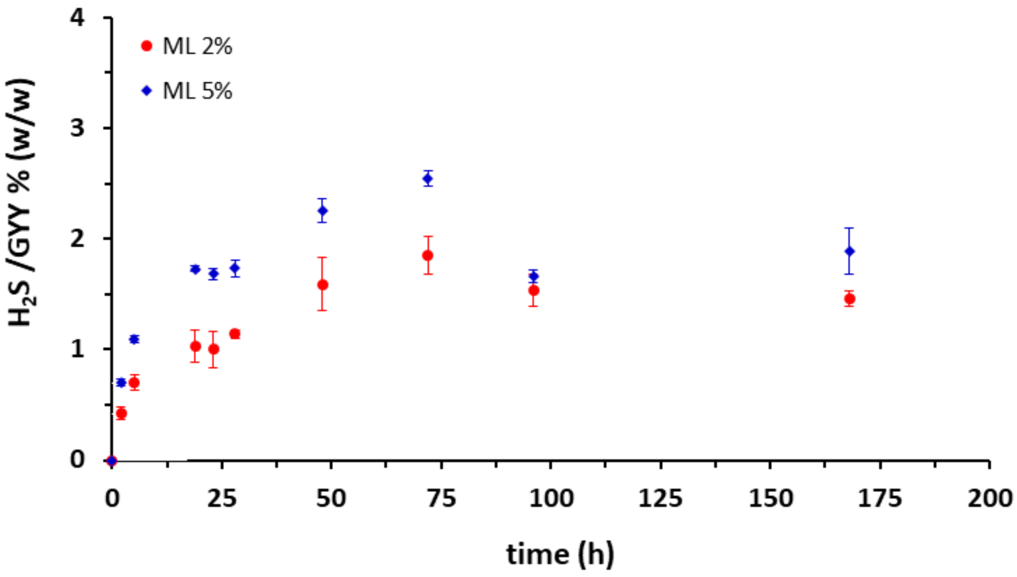

Figure S5. H_2S-release from sterilized multi-layer samples from 0 h to 168 h reported as percentage with respect to the amount of GYY in the scaffold (μg of H_2S/μg of GYY) %.

5. Cell viability evaluation

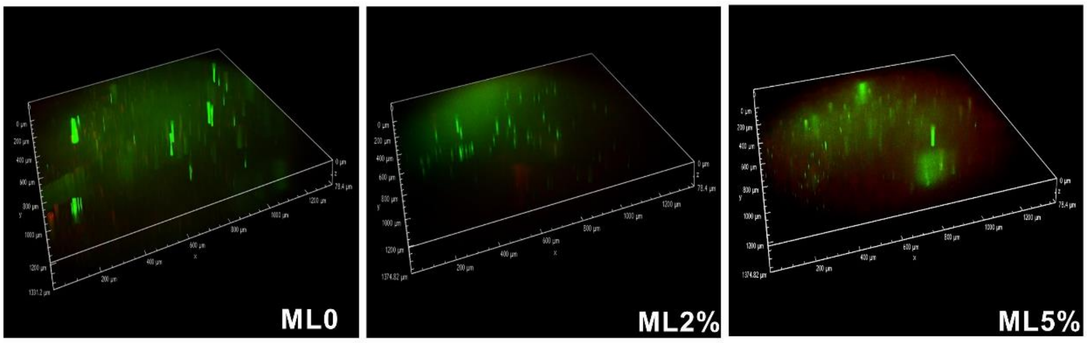

Figure S6. Cell viability evaluated by Calcein AM/Ethidium homodimer staining. Human MSCs were seeded on the scaffolds and stained after 72 h in culture. Panels show one angled-view representative picture taken as Z-stack of 28 consecutive layers with a thickness of 2.8 μm, for a total depth of 78.4 μm across each scaffold.
